# Ecotoxicological Studies of ZnO and CdS Nanoparticles on *Chlorella vulgaris* Photosynthetic Microorganism in Seine River Water

**DOI:** 10.3390/nano10020227

**Published:** 2020-01-28

**Authors:** Alice da Rocha, Nicolas Menguy, Claude Yéprémian, Alain Couté, Roberta Brayner

**Affiliations:** 1 Université de Paris, ITODYS, UMR 7086, CNRS, 15 rue Jean-Antoine de Baïf, F-75205 Paris CEDEX 13, France; alice.da.rocha13@gmail.com; 2 Sorbonne Université, Institut de Minéralogie et de Physique des Milieux Condensés UMR CNRS 7590, Institut de Recherche pour le Développement 4, place Jussieu, 75005 Paris, France; Nicolas.Menguy@impmc.upmc.fr; 3 Muséum National d’Histoire Naturelle, UMR 7245 CNRS, USM 505, Département Régulations, Développement, et Diversité Moléculaire (RDDM), 12 rue Buffon, F-75005 Paris, France; yep@mnhn.fr (C.Y.); acoute@mnhn.fr (A.C.)

**Keywords:** polyol process, CdS, ZnO, ecotoxicology, *Chlorella vulgaris*

## Abstract

Seine river water was used as natural environmental medium to study the ecotoxicological impact of ZnO and CdS nanoparticles and Zn^2+^ and Cd^2+^ free ions using *Chlorella vulgaris* as a biological target. It was demonstrated by viability tests and photosynthetic activity measurements that free Zn^2+^ (*IC*_50_ = 2.7 × 10^−4^ M) is less toxic than free Cd^2+^ and ZnO nanoparticles (*IC*_50_ = 1.4 × 10^−4^ M). In the case of cadmium species, free Cd^2+^ (*IC*_50_ = 3.5 × 10^−5^ M) was similar to CdS nanoparticles (CdS-1: *IC*_50_ = 1.9 × 10^−5^ M and CdS-2: *IC*_50_ = 1.9 × 10^−5^ M), as follows: CdS > Cd^2+^ > ZnO > Zn^2+^. Adenosine-5’-triphosphate (ATP) assay and superoxide dismutase (SOD) enzymatic activity confirmed these results. Transmission electron microscopy (TEM), coupled with energy-dispersive X-ray spectroscopy (EDS), confirmed the internalization of CdS-1 nanoparticles after 48 h of contact with *Chlorella vulgaris* at 10^−3^ M. With a higher concentration of nanoparticles (10^−2^ M), ZnO and CdS-2 were also localized inside cells.

## 1. Introduction

Currently, there is great interest in understanding the biology and environmental consequences of manufactured materials [1,2,3,4,5,6,7]. If we can understand this, we can improve the performance of these materials. In addition, research in this field can only improve the environmental conditions.

Manufactured materials can be released into the environment, impacting living organisms. Despite their worldwide use, no research has focused the degradation of manufactured materials [5,6]. In addition, many studies have focused on the impact of these materials on plants and other living organisms and their possible entry into the food chain [8,9,10,11,12].

The nanoparticles of ZnO and CdS have been used in many fields, such as the elaboration of sunscreens, pigments, and cottages, as well as optico-electronic systems [13]. In all cases, physicochemical studies are essential to understand the behavior of these materials, as well as their uptake and distribution inside microorganisms and their interaction with pollutants. For an environmental risk assessment of nanoparticles, both exposure in the environment (dissolution/aggregation) and hazards, such as toxicity, need to be taken into account.

**Figure 1:**
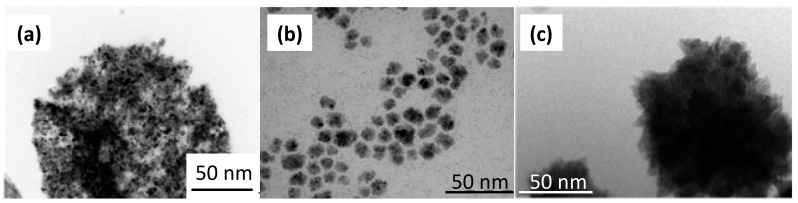
Transmission electron microscopy (TEM) images of (**a**) ZnO, (**b**) CdS-1, amd (**c**) CdS-2 after contact with Seine river water.

To perform ecotoxicological tests, it was necessary to find a natural environment which was rich enough in nutriments to cultivate microalgae. Seine river water is very rich in mineral salts [14,15,16,17] for cultivating microorganisms. Its pH is high (8.01) compared to the physiological pH (7.4), but the first tests with *Chlorella vulgaris* have been promising. ZnO nanoparticles aggregate in the Seine water, while CdS nanoparticles remain isolated [14,15,16,17]. Figure 1 shows micrographs of nanoparticles after contact with seine water.

In this study, we investigated the fate of ZnO and CdS nanoparticles in Seine river water (Paris, France) in the presence of *Chlorella vulgaris* microalgae. We analyzed the physicochemical properties of the nanoparticles and also the microalgae behavior after contact with ZnO and CdS nanoparticles in Seine river water.

## 2. Materials and Methods

### 2.1. Cell Cultures

*Chlorella vulgaris* green algae came from Museum National d’Histoire Naturelle (MNHN) Culture Collection. *Chlorella vulgaris* was grown in 275-mL (or 75 cm^2^) flasks with a 0.22-µm vented plug seal cap in sterile Bold’s basal medium (BB medium) at a pH of 7 or in the Seine river water sterilized using a 0.22-µm filter (cellulose acetate filter, Millipore, USA with a pH of 8.01. *Chlorella vulgaris* was grown at a controlled temperature of 20.0 ± 0.5 °C and luminosity (50–80 μmoL m^−2^ s^−1^ photosynthetic photon flux (PPF). Filtered Seine river water was stored at 4 °C for experiments and constituted our reference interaction medium. Seine river water was collected near Paris Diderot University, Paris, France (GPS: 48.831039° N, 2.381709° E).

### 2.2. Nanoparticles Characterization

Hexagonal (hcp) and face-centered cubic (fcc) CdS (CdS-1 and CdS-2, respectively) and hexagonal (hcp) ZnO nanoparticles were synthesized by the polyol method [4,5,6,7,14]. CdS-1 (hc structure) nanoparticles presented a narrow size distribution with 2-nm diameter and *S_g_* = 67 m^2^ g^−1^. CdS-2 (fcc structure) were agglomerated with more than 100-nm length with heterogeneous diameter and specific surface area (*S_g_*) = 10 m^2^ g^−1^. ZnO (hcp) nanoparticles were anisotropic and presented a 50-nm length and 15-nm diameter and *S_g_* = 54 m^2^ g^−1^ [14].

### 2.3. Cells Concentration/Cells Viability

Cellometer Auto X4 equipment (France) calculated cell concentration and % viability simultaneously for cultured cells stained with trypan blue (1/10% v/v).

### 2.4. Growth Rate

The growth rate (*µ*) was calculated from the change in the cell concentration between the third and the fourth days using the formula: *µ* = (ln*N*_2_ − ln*N*_1_)/(*t*_2_ − *t*_1_); (*N*: Cell concentration, *t*: Time (day)).

### 2.5. PAM Measurements

The photosynthetic activity of microalgae was measured using the pulse-amplitude modulation (PAM) method with a Handy PEA (Hansatech instruments, Germany) fluorometer. This method uses a pulse saturation mode in which the microalgae receives a short beam of light that will saturate the photosystem II (PS II). This process eliminates the photochemical quenching, which then reduces the maximum yield. The fluorescence ratio (*F_v_*/*F_m_*) can therefore be measured.

### 2.6. Electron Microscopy

#### 2.6.1. Transmission Electron Microscopy (TEM) Analyses

During this study, we used two types of TEM equipment: (i) Biomass transmission electron microscopy (TEM) imaging was performed with a Hitachi H-700 (Tokyo, Japan) operating at 75 kV equipped with a Hamatsu camera; (ii) TEM and TEM-EDX observations were carried out on a JEOL 2100F microscope (Tokyo, Japan) operating at 200 kV, equipped with a field emission gun, a high-resolution UHR pole piece, and a Gatan US4000 CCD camera. The particle size distribution was obtained from the TEM images using a digital camera and the elemental mapping were performed using X-ray energy dispersive spectroscopy (XEDS) analyses using a JEOL detector coupled with a scanning TEM device. Using the three window technique Energy Filtered TEM (EFTEM) was performed with a Gatan GIF 2001 imaging filter (Tokyo, Japan).

Energy Dispersive X-ray (EDX) microanalysis is a technique used for identification of the elemental composition of nanoparticles inside cells. EDX was used to confirm the distribution and composition of the nanoparticles through spectrum and elemental mapping.

For TEM and TEM-EDX studies, the microalgae were fixed with a mixture containing 2% glutaraldehyde and picric acid in a phosphate Sorengën buffer (0.1 M, pH 7.4). Cells were contrasted with 0.5% of osmium tetraoxyde. Dehydration was then achieved in a series of ethanol baths, and the samples were processed for flat embedding in a Spurr resin or dried by critical point for scanning electron microscopy (SEM) observations. Ultrathin sections of samples in the resin were made using a Reicherd E Young Ultracut ultramicrotome (Leica, Wetzlar, Germany).

#### 2.6.2. Scanning Electron Microscopy (SEM) Analyses

For SEM studies, the samples were imaged by a Zeiss Supra 4 Scanning Electron Microscope (Oberkochen, Germany) equipped with an in-lens detector. For imaging, very low excitation voltage (1 kV) and a small sample-detector distance (3.3 or 4.5 mm) were used. Under these experimental conditions, charging effects were minimal.

### 2.7. ATP Assay

Adenosine-5’-triphosphate (ATP) levels of the samples were improved by a luciferase-luciferin enzymatic assay kit BacTiter-Glo^TM^ from Promega (France). The BacTiter-Glo^TM^ reagent is directly added to microalgae cells in medium and triggers cell lysis. The luminescence can be measured without washing cells or removing medium. ATP concentration was performed using a standard curve. All experiments were done in triplicate.

### 2.8. Superoxide Dismutase (SOD) Enzymatic Activity

SOD enzymatic activity measurements were performed using 19,160 SOD determination kit from Sigma-Aldrich (France). SOD enzymatic activity was determined using colorimetric measurements at 440 nm.

### 2.9. Statistical Analysis

All experiment were made in triplicate. The data were analyzed by one-way analyses of variance (ANOVAs). The level of significance in all comparisons was *p* < 0.05.

## 3. Results and Discussion

**Figure 2:**
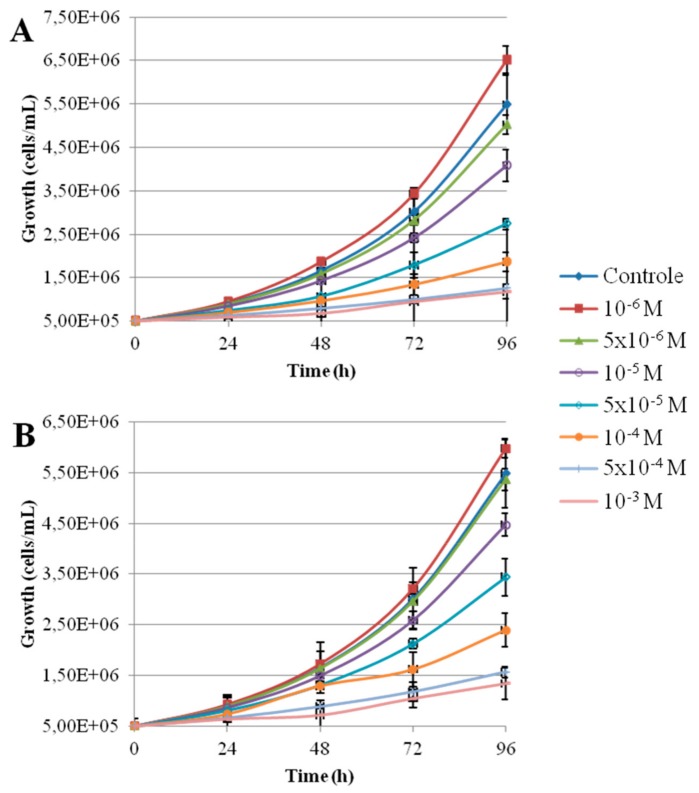
Growth rate of *Chlorella vulgaris* as a function of time after contact with Zn^2+^ free ions and ZnO nanoparticles in Seine river water: (**A**) Zn^2+^, (**B**) ZnO.

**Figure 3:**
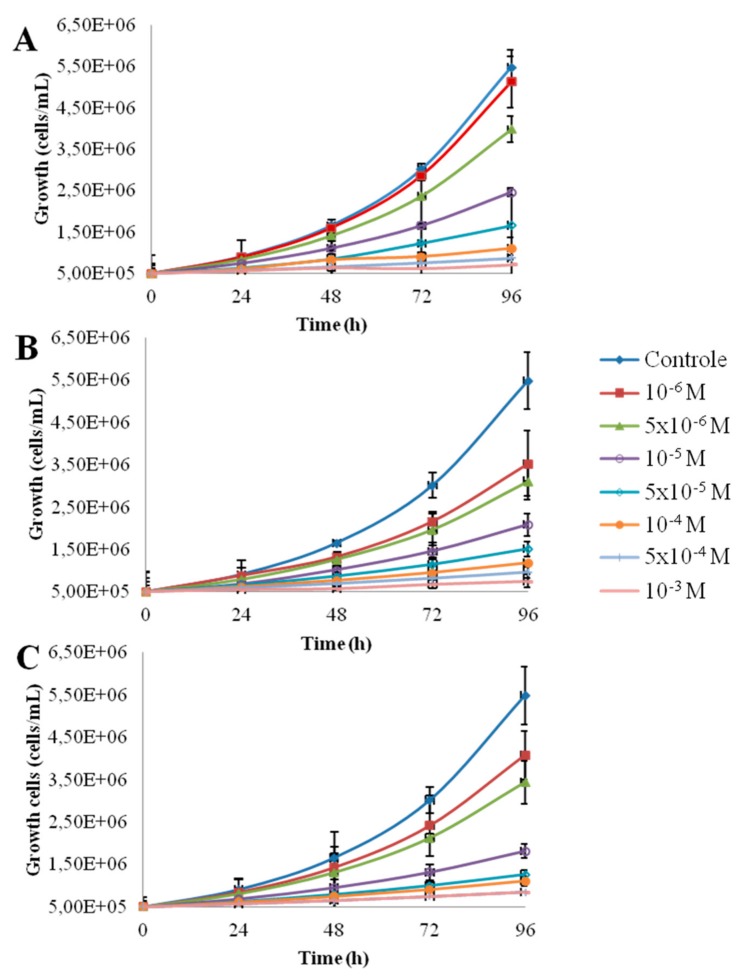
Growth rate of *Chlorella vulgaris* as a function of time after contact with Cd^2+^ free ions and CdS-1 and CdS-2 nanoparticle in Seine river water: (**A**) Cd^2+^, (**B**) CdS-1, (**C**) CdS-2.

**Figure 4:**
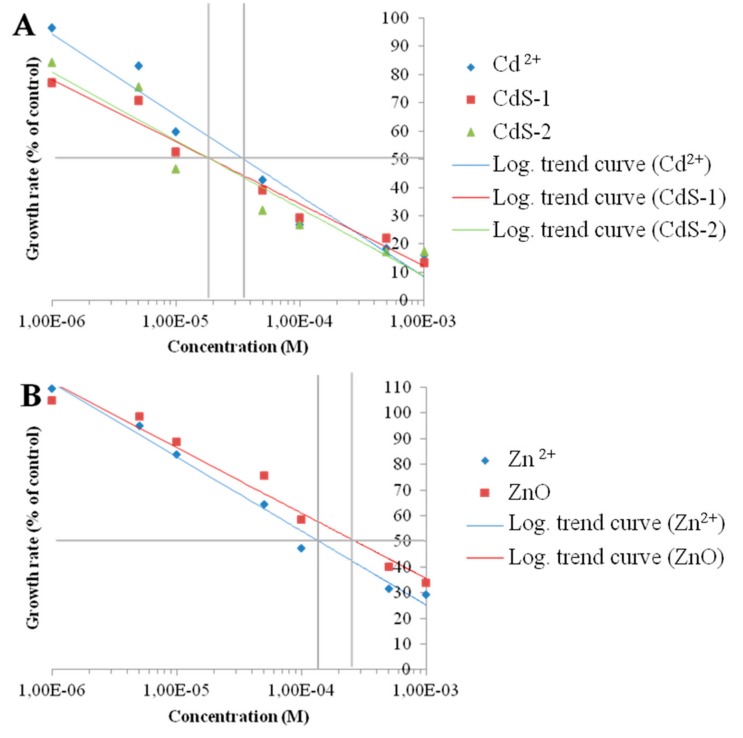
*IC*_50_ logarithmic trend curves as a function of growth rate. (**A**) Zn^2+^ and ZnO and (**B**) Cd^2+^, CdS-1, and CdS-2 are expressed as % of control. Growth rate was determined between the third and the fourth days.

Figure 1 shows the morphology of the nanoparticles using TEM analysis after contact with Seine river water. ZnO and CdS-2 nanoparticles were agglomerated when CdS-1 were very well-dispersed. Figure 2 and Figure 3 show the growth rate for *Chlorella vulgaris* as a function of the time after contact with ZnO, CdS-1, and CdS-2 nanoparticles and Zn^2+^ and Cd^2+^ free ions in Seine river water. For ZnO nanoparticles and Zn^2+^ free ions, a high toxicological impact on cell growth was observed between 10^−4^ and 10^−3^ M. On the other hand, for CdS-1, CdS-2 nanoparticles and Cd^2+^ free ions, the toxicological impact on cell growth start at 10^−5^ M. Figure 4 presents the logarithmic trends curves used to determine the *IC*_50_ by extrapolation for ZnO, CdS-1, and CdS-2 nanoparticles and Zn^2+^ and Cd^2+^ free ions after contact with *Chlorela vulgaris* green microalgae. For ZnO nanoparticles and Zn^2+^ free ions, the *IC*_50_ was 1.4 × 10^−4^ M and 2.7 × 10^−4^ M, respectively. ZnO nanoparticles were most toxic than Zn^2+^ free ions. These results were very close to ZnO nanoparticles and Zn^2+^ free ions after contact with *Pseudokirchineriella subcaptata* green microalgae (*IC*_50_ = 60 μg/L) [18] and were less toxic than ZnO nanocrystals after contact with cancer cell lines (*IC*_50_ = 150 μM) [19]. For CdS-1, CdS-2, and Cd^2+^ free ions, the *IC*_50_ was 1.9 × 10^−5^ M, 1.9 × 10^−5^ M, and 3.5 × 10^−5^ M, respectively. In this case, Cd^2+^ free ions were similar to CdS nanoparticles. These results were very close to amino acid-modified *β*-CD-coated CdSe/CdS quantum dots after contact with HeLa cells (*IC*_50_ = 68.6 μg/mL) [20]. For ZnO, CdS-1, and CdS-2 nanoparticles and Zn^2+^ and Cd^2+^ free ions, the ecotoxicological impact on *Chlorella vulgaris* growth rate was observed as follows: CdS > Cd^2+^ > ZnO > Zn^2+^.

**Figure 5:**
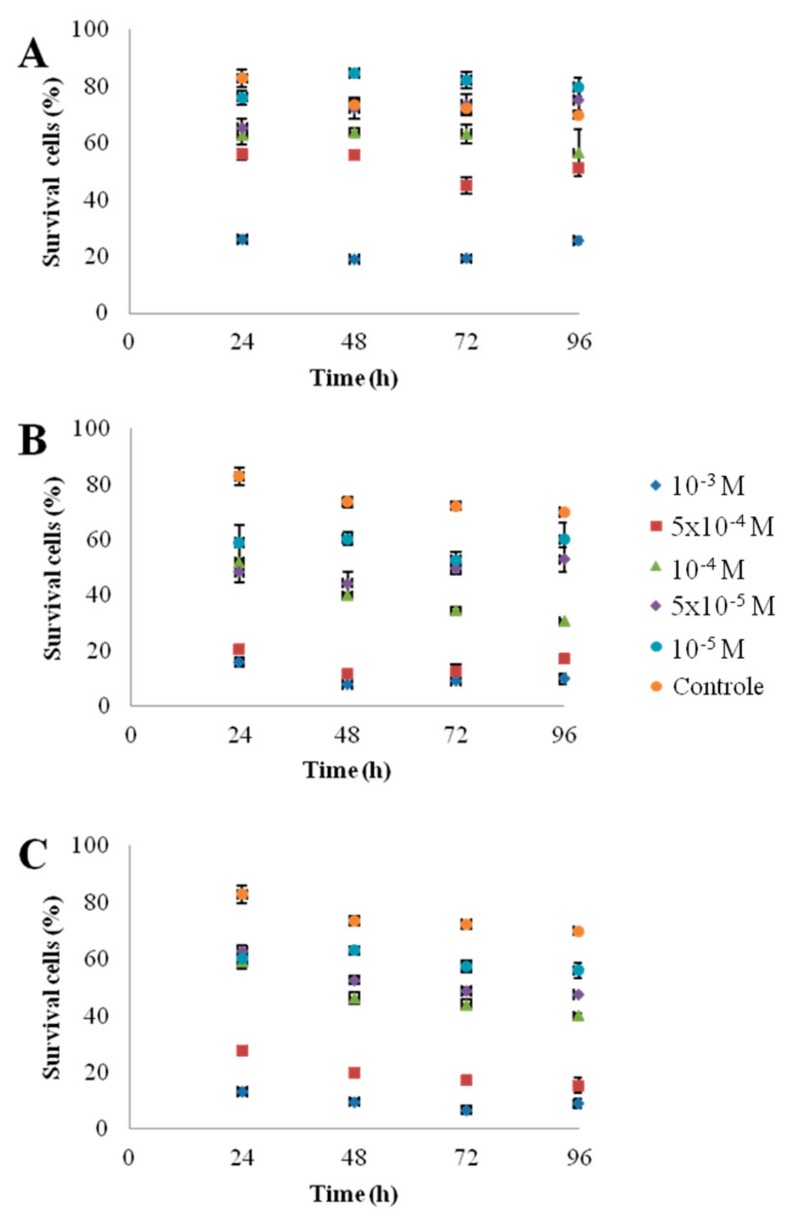
*Chlorella vulgaris* viability as a function of time after contact with nanoparticles in Seine river water: (**A**) ZnO, (**B**) CdS-1, (**C**) CdS-2.

**Figure 6:**
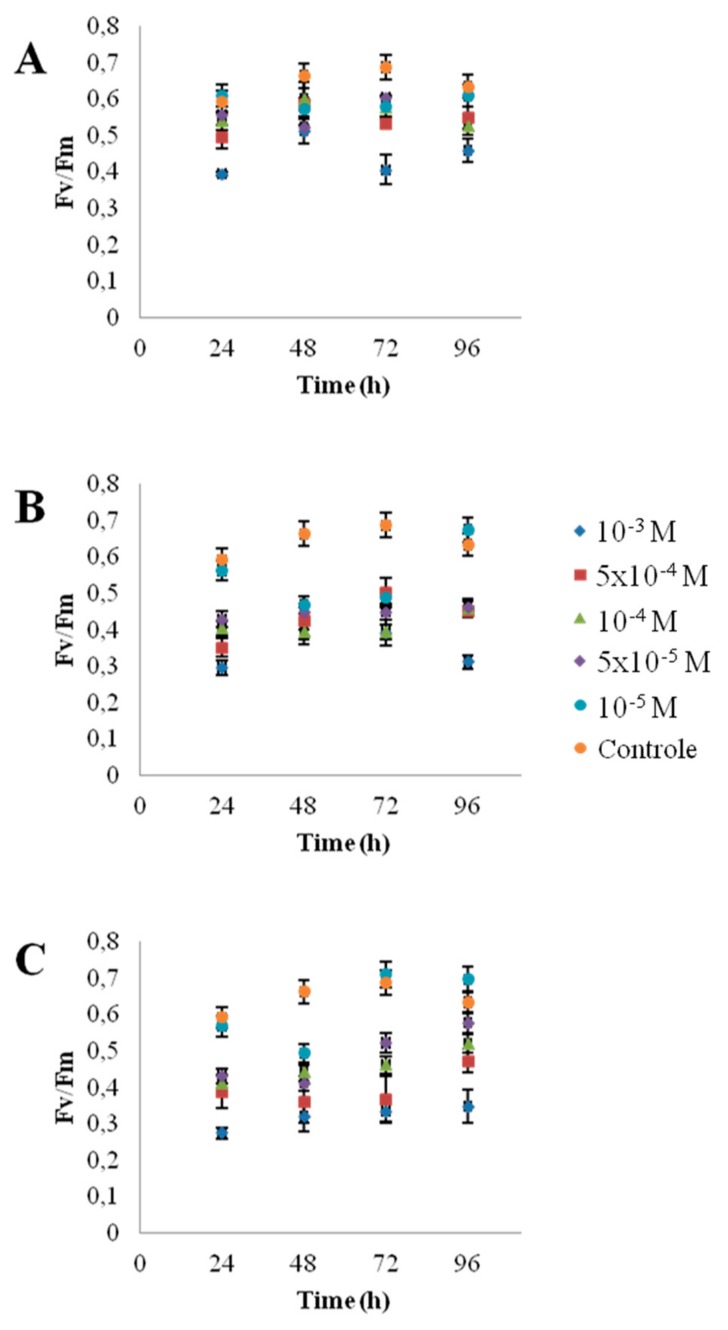
Photosynthetic activities of *Chlorella vulgaris* as a function of time after contact with ZnO, CdS-1 and CdS-2 nanoparticles in Seine river water: (**A**) ZnO, (**B**) CdS-1, (**C**) CdS-2.

**Figure 7:**
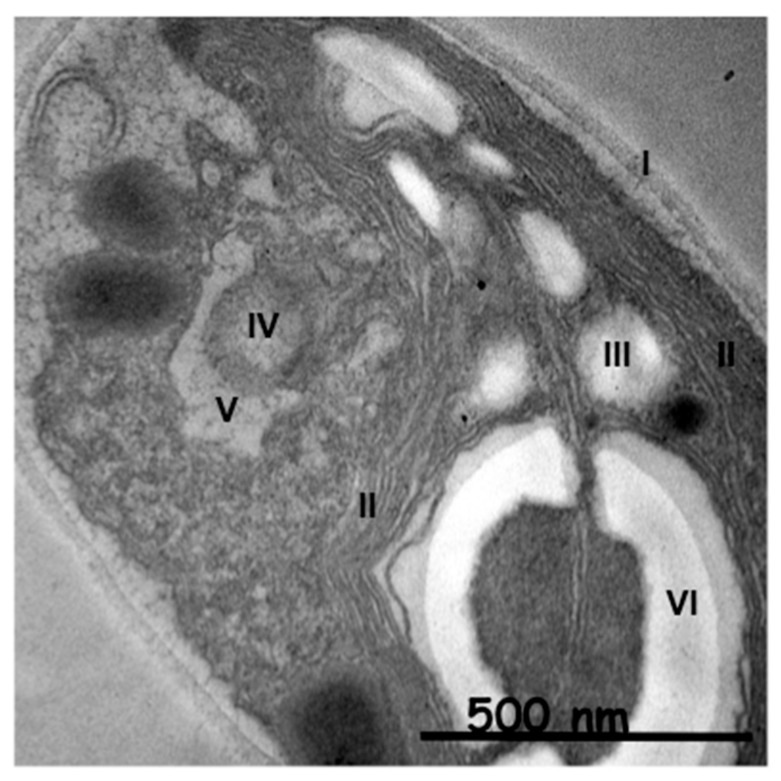
TEM micrograph of *Chlorella vulgaris* ultrastructure in Seine river water: (I) Cell membrane, (II) thylakoids, (III) paramylon vesicle, (IV) nucleus, (V) nuclear membrane, (VI) pyrenoid.

**Figure 8:**
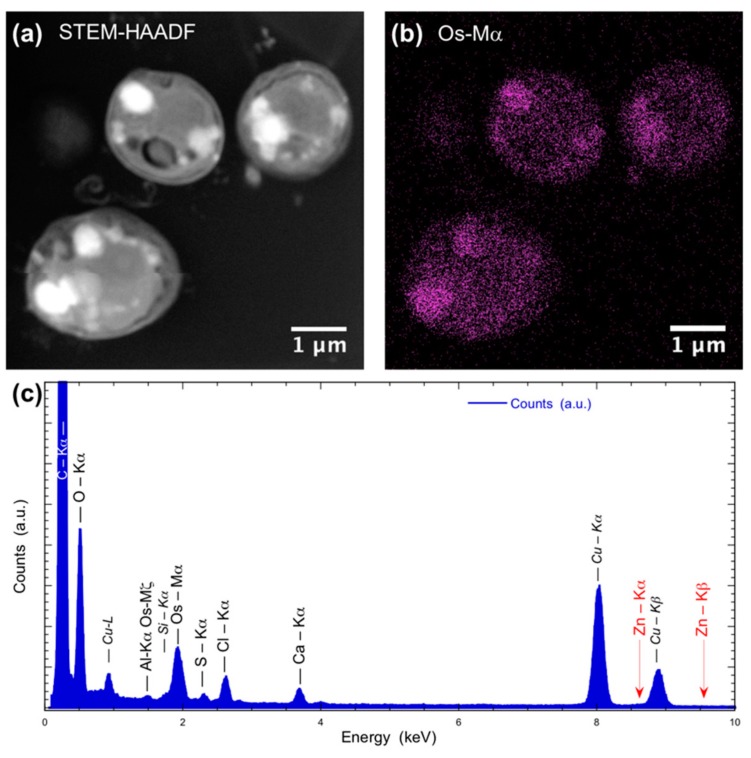
(**a**) STEM-HAADF and (**b**) STEM-XEDS images of *Chlorella vulgaris* ultrastructure after contact with ZnO nanoparticles (10^–3^ M) in Seine river. Bright areas within the cell are due to the accumulation of contrasting agent OsO_4_. (**c**) XEDS spectrum showing the absence of Zinc.

**Figure 9:**
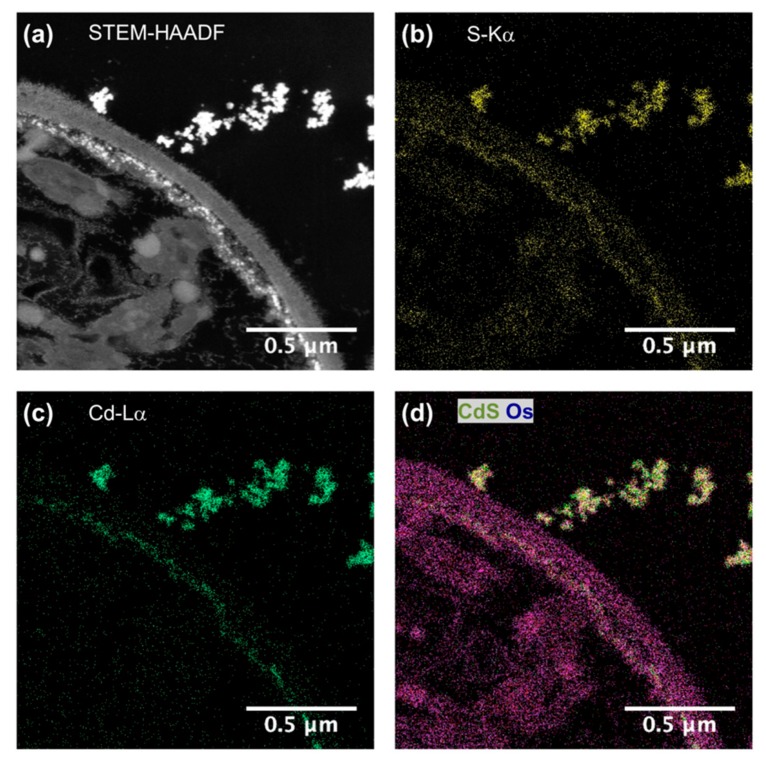
(**a**) STEM-HAADF and (**b**–**d**) STEM-XEDS images of *Chlorella vulgaris* ultrastructure after contact with CdS–1 nanoparticles (10^–3^ M) in Seine river.

**Figure 10:**
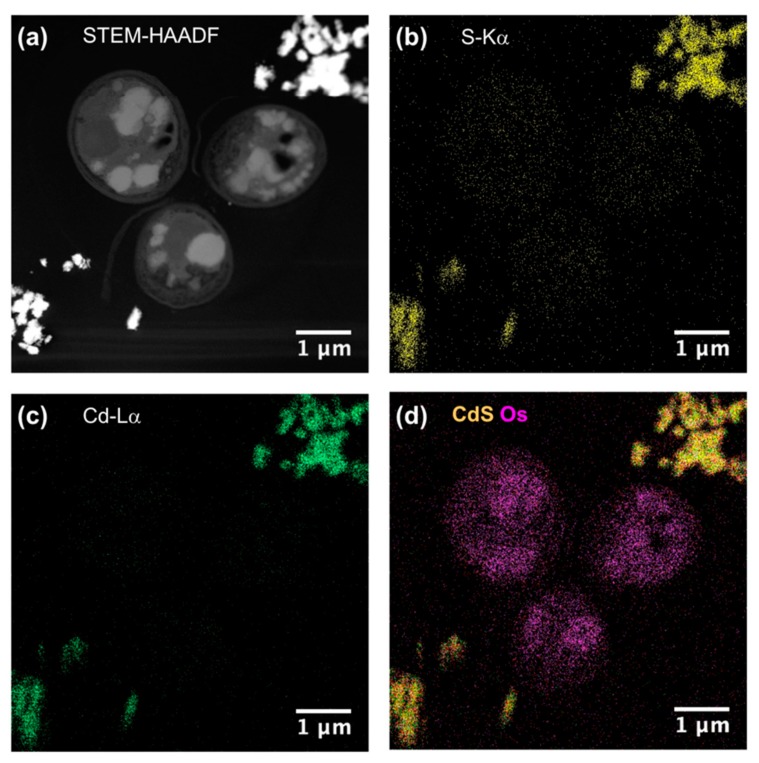
(**a**) STEM-HAADF and (**b**–**d**) STEM-XEDS images of *Chlorella vulgaris* ultrastructure after contact with CdS–1 nanoparticles (10^–3^ M) in Seine river.

**Figure 11:**
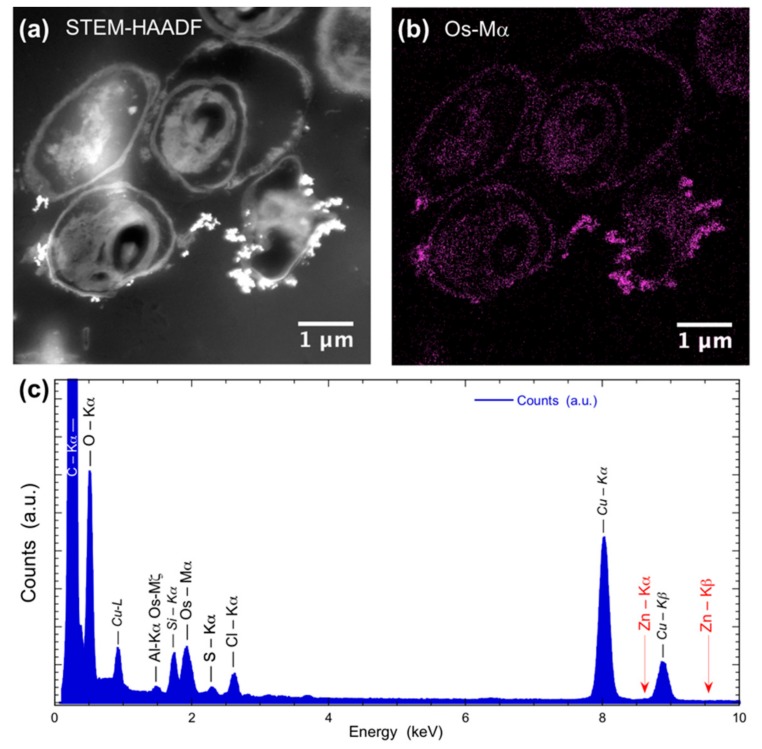
(**a**) STEM-HAADF and (**b**) STEM-XEDS images of *Chlorella vulgaris* ultrastructure after contact with ZnO nanoparticles (10^–3^ M) in Seine river. Bright areas within the cell are due to the accumulation of contrasting agent OsO_4_. (**c**) XEDS spectrum showing the absence of Zinc.

**Figure 12:**
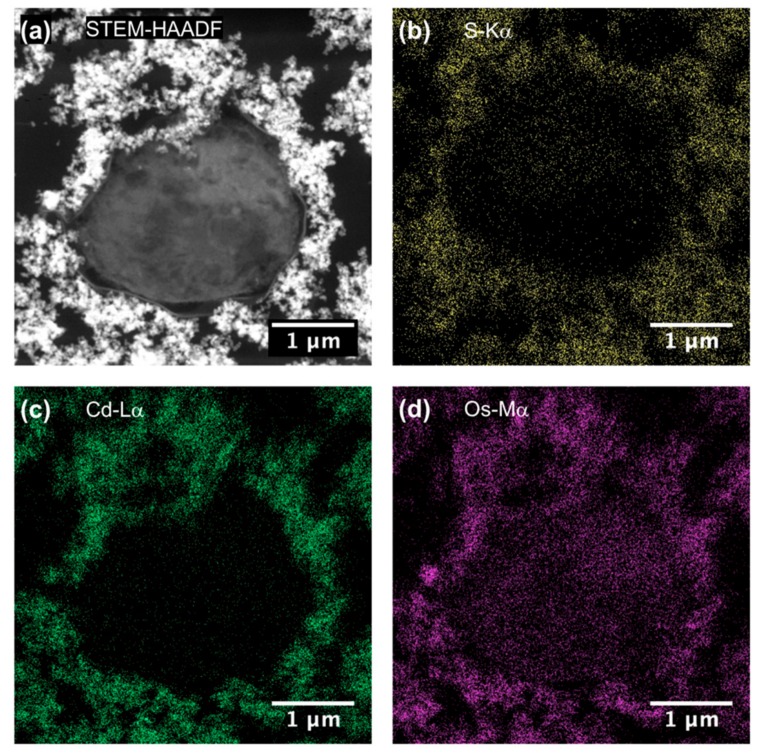
(**a**) STEM-HAADF and (**b**–**d**) STEM-XEDS images of *Chlorella vulgaris* ultrastructure after contact with CdS–1 nanoparticles (10^–2^ M) in Seine river.

**Figure 13:**
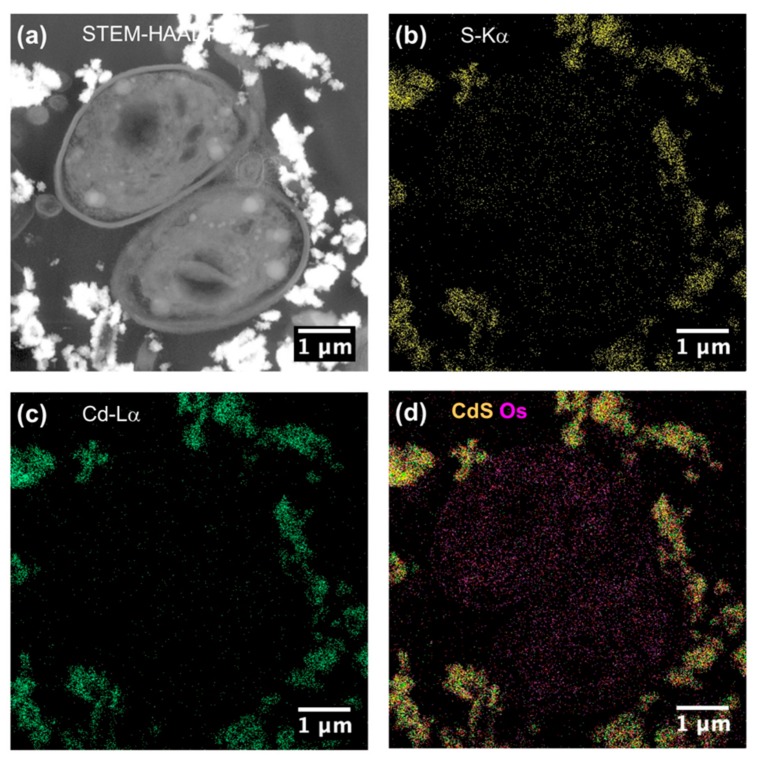
(**a**) STEM-HAADF and (**b**–**d**) STEM-XEDS images of *Chlorella vulgaris* ultrastructure after contact with CdS–2 nanoparticles (10^–2^ M) in Seine river.

**Figure 14:**
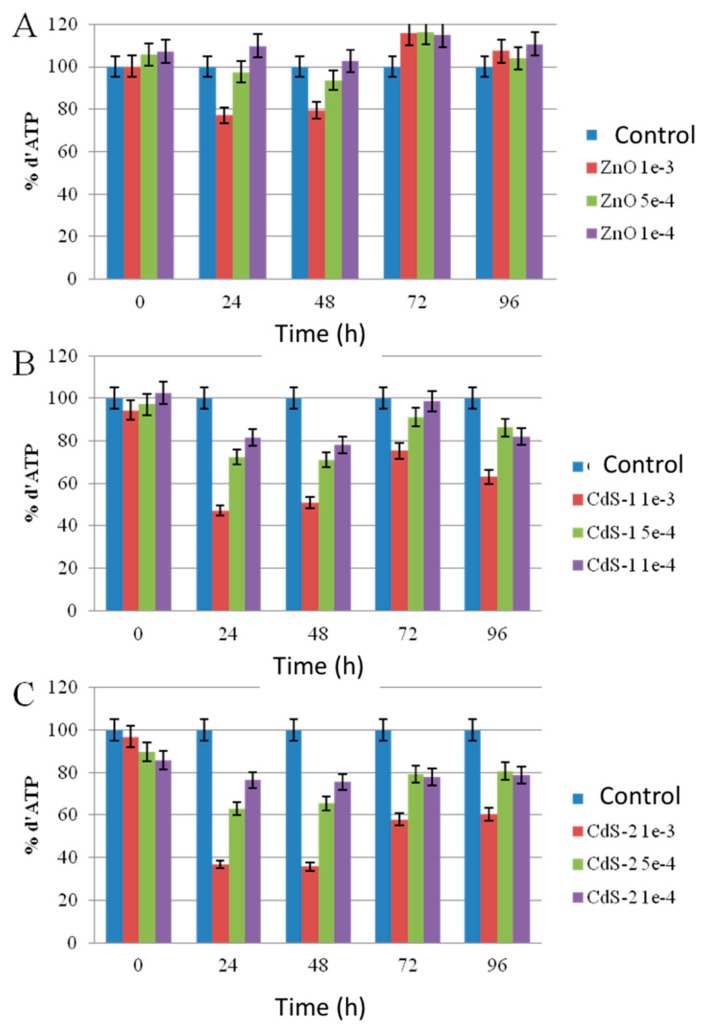
Evolution of the adenosine-5’-triphosphate (ATP) produced by *Chlorella vulgaris* in the presence of (**A**) ZnO, (**B**) CdS-1, and (**C**) CdS-2 nanoparticles in Seine river water as a function of nanoparticles concentration and time.

To be sure that observed growth rate corresponded to the viability of *Chlorella vulgaris* in Seine river water in the presence of nanoparticles, a live/dead test was conducted using trypan blue dye. Trypan blue was used to selectively color dead cells blue. Live cells or tissues with intact cell membranes were not colored. Figure 5 shows the cellular viability of *Chlorella vulgaris* in Seine river water after contact with the nanoparticles. In all cases, a decrease of cell viability was observed as a function of time. For ZnO nanoparticles, 10^−3^ M concentration was very toxic, with % of survival near 20%. For the other concentrations, the % of survival varied from 60% to 80%. It was shown for ZnO nanoparticles that the % of survival after contact with *Anabaena flos-aquae* cyanobacteria was near 80% [6]. On the other hand, after contact with *Euglena gracilis* microalgae, the % of survival decreased to 5% [6]. In the case of *Anabaena flos-aquae*, the presence of exopolysaccharides avoided particle internalization. Moreover, ZnO nanoparticles were internalized by endocytosis in the case of *Euglena gracilis,* which did not present exopolysaccharides [6]. For CdS-1 and CdS-2 nanoparticles, the % of survival was near 20% between 10^−3^ and 5 × 10^−4^ M, showing that these nanoparticles were more toxic than ZnO. These results were in agreement with growth rate tests (Figure 2, Figure 3 and Figure 4). The photosynthetic activities of *Chlorella vulgaris* as a function of time after contact with ZnO, CdS-1, and CdS-2 nanoparticles in Seine river water are shown in Figure 6. *Chlorella vulgaris*, in Seine river water, presented a stable photosynthetic activity (*F_v_*/*F_m_* = 0.7) for more than two months. A photosynthetic activity decrease was observed as a function of nanoparticle concentrations in all cases. No cell death was observed. When compared with *Anabaena flos-aquae* cyanobacteria and *Euglena gracilis* microalgae, *Chlorella vulgaris* green microalgae showed high resistance against ZnO and CdS nanoparticles [6,7]. *Anabaena flos-aquae*, after contact with ZnO nanoparticles (10^−3^ M), presented first a progressive decrease of photosynthetic activity, followed by an adaptation period and, finally, a progressive increase to a normal activity [6]. On the other hand, in the presence of CdS nanoparticles (10^−3^ and 5 × 10^−4^ M), the photosynthetic activity decreased followed by cell death [7]. For *Euglena gracilis*, after contact with ZnO and CdS nanoparticles, the decrease of photosynthetic activities was always followed by cell death [6,7]. Figure 7 presents *Chlorella vulgaris* ultrastructure after contact with Seine river water. The cell membrane integrity was preserved. The chloroplast presented thylakoids membranes where photosynthesis took place, as well as paramylon vesicles and pyrenoid associated with synthesis and storage of starch. Consequently, cell ultrastructure was preserved. Figure 8, Figure 9, Figure 10, Figure 11, Figure 12 and Figure 13 show TEM-EDX micrographs of *Chlorella vulgaris* ultrastructure after contact with ZnO, CdS-1, and CdS-2 nanoparticles at 10^−3^ and 10^−2^ M concentrations, in Seine river water. At 10^−3^ M, only CdS-1 nanoparticle internalization was observed (Figure 8). The nanoparticles were found in the periplasm. At 10^−2^ M, nanoparticle internalization was observed after contact with ZnO, CdS-1, and CdS-2 nanoparticles, and was only observed in periplasm (Figure 11, Figure 12 and Figure 13). Moreover, at 10^−2^ M, we observed changes in cell morphology: An increase of paramylon concentration (which may have been caused by cell stress), cell membrane disruption that helps nanoparticles internalization. To better understand the toxicological impact of ZnO and CdS nanoparticles on *Chlorella vulgaris* green microalgae metabolism in Seine river water, two biochemical tests were performed: An ATP assay and superoxide dismutase (SOD) enzymatic activity. Figure 14 shows the evolution of ATP produced by *Chlorella vulgaris* in the presence of ZnO, CdS-1 and CdS-2 nanoparticles in Seine river water as a function of nanoparticles concentration and time. For ZnO nanoparticles at 5 × 10^−4^ and 10^−4^ M, an increase of ATP production was observed as a function of the time. Moreover, for 10^−3^ M concentration, a decrease of ATP production was observed at 24 h and 48 h. Between 72 h and 96 h, the ATP production remained constant. The behavior of CdS-1 and CdS-2 was very close. For CdS-1 and CdS-2, the inhibition of ATP production as a function of the nanoparticles concentration was also observed. However, in this case, the decrease of ATP production for 10^−3^ M nanoparticles concentration was more important at 24 h. The impact of ATP production was more important after contact with CdS nanoparticles. In addition, the toxicity of CdS-2 was close to CdS-1. For *Chlorella vulgaris* ATP production, the nanoparticle impact was observed as follows: CdS-2 ≅ CdS-1 > ZnO.

**Figure 15:**
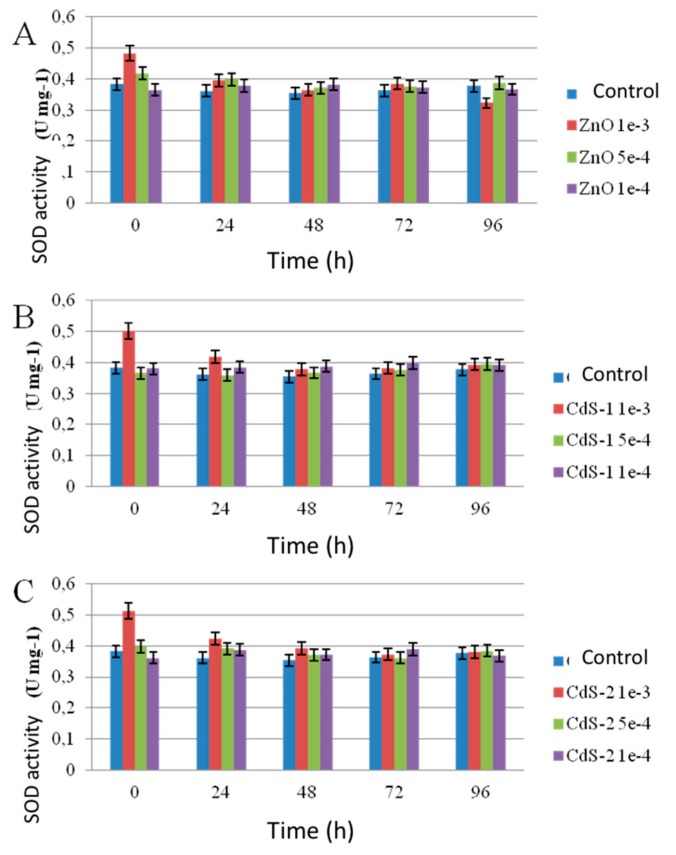
Superoxide dismutase (SOD) activity after contact with *Chlorella vulgaris* in the presence of (**A**) ZnO, (**B**) CdS-1, and (**C**) CdS-2 in Seine river water as a function of the nanoparticles concentration and time.

These results are in agreement with growth rate, live/dead tests, and photosynthetic activity. It has been shown that oxidative stress metabolites in plants, namely microalgae, can be used as biomarkers in the ecotoxicological study [21]. The reactivity of oxygen species (ROS) that promote the oxidative stress in microorganisms in the presence of nanoparticles was measured using the enzymatic activity of superoxide dismutase (SOD). Figure 15 presents SOD enzymatic activities of *Chlorella vulgaris* in the presence of ZnO, CdS-1, and CdS-2 nanoparticles in Seine river water as a function of nanoparticles concentration and time. In all cases, an increase of SOD enzymatic activity was observed at 10^−3^ M nanoparticles concentration in 24 h. After this time, no SOD enzymatic activity was observed. In this case, *Chlorella vulgaris* adapt its metabolism after 24 h of contact with the nanoparticles. The increase of SOD activity at 10^−3^ M of nanoparticles concentration in 24 h was observed as follows: CdS-2 ≅ CdS-1 > ZnO.

These results are in agreement with ATP assay.

## 4. Conclusions

In this work, Seine river water was used as natural environmental medium to study the ecotoxicological impact of ZnO and CdS nanoparticles and Zn^2+^ and Cd^2+^ free ions using *Chlorella vulgaris* as biological target. It was demonstrated by growth rate tests that the toxicity of the nanoparticles and free ions was:

CdS > Cd^2+^ > ZnO > Zn^2+^

These results were in agreement with live/dead tests and photosynthetic activity measurements. TEM analyses showed CdS-1 particle internalization (only in the periplasm) at 10^−3^ M of nanoparticles concentration. On the other hand, at 10^−2^ M of nanoparticles concentration, internalization was observed using all nanoparticles. To better understand the behavior of *Chlorella vulgaris* microalgae in the presence of ZnO, CdS-1 and CdS-2 nanoparticles biochemical tests were also made as a function of nanoparticles concentration and time. ATP assay and SOD enzymatic activity showed that CdS nanoparticles were more toxic for *Chlorella vulgaris* than ZnO nanoparticles as follows:

CdS-2 ≅ CdS-1 > ZnO

These results are in agreement with growth rate and live/dead tests and photosynthetic activity measurements.
